# Rapid and Green Analytical Method for the Determination of Quinoline Alkaloids from *Cinchona succirubra* Based on Microwave-Integrated Extraction and Leaching (MIEL) Prior to High Performance Liquid Chromatography

**DOI:** 10.3390/ijms12117846

**Published:** 2011-11-14

**Authors:** Anne-Sylvie Fabiano-Tixier, Abdelhakim Elomri, Axelle Blanckaert, Elisabeth Seguin, Emmanuel Petitcolas, Farid Chemat

**Affiliations:** 1 UMR 408 INRA, Safety and Quality of Plant Products, University of Avignon, 84000 Avignon, France; E-Mails: emmanuel.petitcolas@univ-avignon.fr (E.P.); farid.chemat@univ-avignon.fr (F.C.); 2 CNRS UMR 6014, C.O.B.R.A., UFR Medicine-Pharmacy, University of Rouen, 76183 Rouen, France; E-Mails: Hakim.Elomri@univ-rouen.fr (A.E.); axelle.blanckaert@univ-rouen.fr (A.B.); elisabeth.seguin@univ-rouen.fr (E.S.)

**Keywords:** *Cinchona* alkaloids, Soxhlet, microwave, extraction, HPLC

## Abstract

Quinas contains several compounds, such as quinoline alkaloids, principally quinine, quinidine, cinchonine and cichonidine. Identified from barks of *Cinchona*, quinine is still commonly used to treat human malaria. Microwave-Integrated Extraction and Leaching (MIEL) is proposed for the extraction of quinoline alkaloids from bark of *Cinchona succirubra*. The process is performed in four steps, which ensures complete, rapid and accurate extraction of the samples. Optimal conditions for extraction were obtained using a response surface methodology reached from a central composite design. The MIEL extraction has been compared with a conventional technique soxhlet extraction. The extracts of quinoline alkaloids from *C. succirubra* obtained by these two different methods were compared by HPLC. The extracts obtained by MIEL in 32 min were quantitatively (yield) and qualitatively (quinine, quinidine, cinchonine, cinchonidine) similar to those obtained by conventional Soxhlet extraction in 3 hours. MIEL is a green technology that serves as a good alternative for the extraction of *Cinchona* alkaloids.

## 1. Introduction

Traditionally used in the Andes (South America) for treating fevers, barks of some plant species popularly named “Quinas” have been known for centuries to possess therapeutic properties. “Quinas” were composed by different plant species belonging to *Cinchona* or *Remijia* genius (Rubiaceae). They were introduced in Europe in the 17th century by Spanish missionaries and became widely used for their antimalarial activity [1].

Several compounds such as phenolic compounds, organic acids and saponosides have been identified from barks of the most studied “Quinas”. More importantly, they are known to contain quinoline alkaloids, principally quinine, quinidine, cinchonine and cinchonidine. First identified from barks of *Cinchona*, quinine is still commonly used to treat human malaria; it remains the drug of reference, because of its broad availability and, contrary to synthetic drugs, the low resistance of *Plasmodium falciparum* to this molecule [2]. Despite the findings of synthetic quinine, *Cinchona* bark remains the principal producer of quinine.

Even though different chromatographic procedures have been developed for the analysis of *Cinchona* alkaloids [3,4], Soxhlet extraction is the reference method for extraction of quinoline alkaloids from *Cinchona* bark powder after treatment with alkali [5]. The Soxhlet extraction was first used for extraction in agricultural chemistry before becoming the most commonly used tool for solid-liquid extraction in many fields like pollutants analysis [6–8], foodstuffs [9–11] and also pharmaceutics [12–14]. Nowadays, Soxhlet apparatus is still common in laboratories and is presented as the standard and reference method for solid-liquid extraction in most cases (ISO 659-1988) [15]. The desired compounds are extracted by an interactive percolation of a fresh solvent. Nevertheless, Soxhlet extraction has some disadvantages such as long operation time required (several hours), evaporation and concentration needed at the end of the extraction, and inadequacy for thermolabile analytes [16].

Microwave energy is known to have a significant effect on the rate of various processes in the chemical and food industry. Much attention has been given to the application of microwave dielectric heating in analytical chemistry because of the reduced analysis time, simplified manipulation and higher purity of the final product. The advantages of using microwave energy as a non-contact heat source for the extraction of analytes from plant materials include: more effective heating, faster energy transfer, reduced thermal gradients, selective heating and reduced equipment size, faster response to process heating control, faster start-up, increased production, and elimination of process steps [17].

All the reported applications have shown that microwave-assisted solvent extraction (MAE) is a viable alternative to conventional techniques for such matrices. The main benefits are the reduction of extraction time, energy and solvent used [18–20]. Applications of MAE in natural product extraction [21,22] or environmental contaminants [23] were reported in recent years. Different plants belonging to the Rubiaceae species have recently been investigated [24]. Since 1998, Luque de Castro *et al*. developed several extraction techniques for microwave-assisted extraction of natural products [25,26]. These systems have been used for the determination of the oil content and the fatty acid composition of oleaginous seeds [27], lipids from sausage products [28], fat from cheese [29] and bakery products [30]. These microwave extraction processes present the advantage of accelerating the whole extraction process. However, no information has been found on the application of MAE for extraction of quinoline alkaloids in *Cinchona*.

The aim of this present study was to investigate Microwave-Integrated Extraction and Leaching (MIEL) ability to extract quinoline alkaloids from bark of *C. succirubra*. A Response Surface Methodology obtained from a multivariate study was used to investigate the performances of MIEL and to study the relevance of factors required during operating extraction. The extraction conditions were optimized in order to obtain an optimum yield. To investigate the potential of MIEL, comparative analysis using high-performance liquid chromatography (HPLC) have also been made with conventional Soxhlet extraction.

## 2. Experimental Section

### 2.1. Apparatus

**Figure 1:**
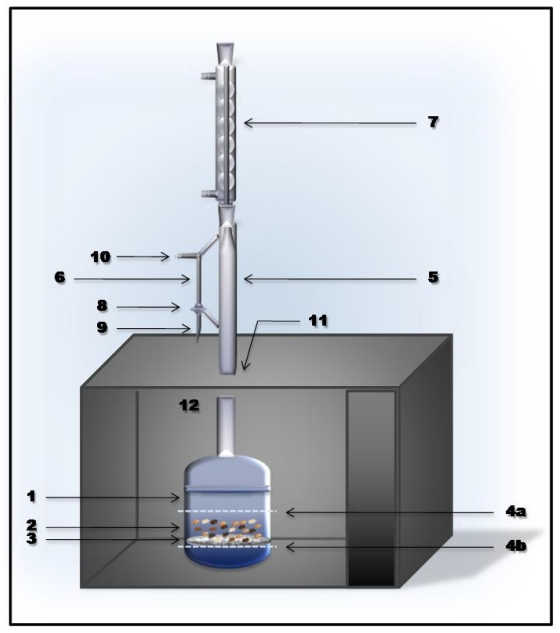
The basic principle of Microwave–Integrated Extraction and Leaching (MIEL). 1: base vessel; 2: solid material; 3: support; 4a: solvent level immersing the sample; 4b: solvent level below the sample; 5: extraction tube; 6: side arm; 7: condenser; 8: 3-way valve; 9: side arm opening to collect solvent; 10: side arm opening to pull a vacuum in the system; 11: opening on upper surface of microwave oven; 12: microwave oven.

The basic principle of the process of microwave-integrated extraction and leaching is illustrated in the Figure 1. MIEL extraction has been performed in Milestone NEOS microwave oven. This is a multimode microwave reactor 2.45 GHz with a maximum delivered power of 1000 W variable in 10 W increments. Temperature was monitored by an external infrared (IR) sensor. During experiments, time, temperature, pressure and power can be controlled.

The base vessel is a traditional glass round-bottom flask. The flask (1) for containing the solid material is a flask suited for microwave reactions. The base vessel contains an inner support (3) for placing the solid material (2) to be extracted. The inner support is a porous support made out of material which absorbs or not microwave radiation. Preferably, the support made of polytetrafluoroethylene (PTFE) is placed at a defined distance above the bottom of the base vessel.

This technique presents the advantage that, after the method has been performed, the solid material (2) placed on said support can easily be separated from the residual solvent which is collected at the bottom of the vessel. The device for carrying out the extraction further comprises an extraction tube (5) which is placed on top of the base vessel. The extraction tube is typically a glass tube. Thus, the microwave oven (12) is provided with an opening on its upper surface (11) such that the extraction tube (5) which is fitted on top of the base vessel may extend from inside the microwave oven (12) to outside. The extraction tube (5) comprises a side arm (6) which is provided with at least one valve and one opening (9). Depending on how the valve is adjusted, the solvent may reflux down the sidearm (6) back into the extraction tube (5) and eventually back into the base vessel (1) or when the valve is adjusted accordingly, the refluxing solvent may be collected from the opening (9). Additionally, the side arm provided with another opening (10) which, depending on the application, may be used to pull a vacuum in the system.

A condenser (7) is placed on top of the extraction tube (5) in order to allow the solvent present in the base vessel to reflux upon microwave irradiation. Refluxing allows the sample to be extracted to be repeatedly percolated, thus increasing the extraction yield. The extraction using the proposed method is carried out by immersing the solid sample material into the vessel containing the solvent under reflux, where it undergoes repeated percolations with the same organic solvent. The four stages of the process are preceded of the preparation of the material.

### 2.2. Reagents and Solutions

The bark powder of Cinchona *succiruba* was purchased by Cailleau (Chemillé, France). Dichloromethane, calcium hydroxide, sodium hydroxide, quinine, quinidine sulfate, cinchonine and cinchonidine were purchased by VWR International (Strasbourg, France). All reagents and solvents used in the HPLC analysis were of analytical grade. Water used in the mobile phase was deionized and filtered through a 0.45 μm membrane.

### 2.3. Sample Preparation

An amount of 15 g of the bark powder was treated with 6 g of calcium hydroxide and 15 mL of sodium hydroxide (5%).

### 2.4. MIEL Procedure

The sample is introduced into a paper filter and placed onto the PTFE filter support (3) and 300 mL of dichloromethane is added in order to immerse the sample (4a). Then, the base vessel is placed in the microwave oven (12) and screwed together with the extraction tube (5). The condenser (7) is placed on the extraction tube and the system is started. The four steps are as follows.

First, the solvent is heated up to the boiling point by microwaves. The solvent vapors penetrate through the sample and the condensation takes place on the condenser. Then, the condensate drips down onto the sample by adjusting the 3-way valve (8). The extraction is performed for 5 min.

Second, the level of the solvent is lowered below the sample (4b) by adjusting the 3-way valve accordingly during 10 min. Third, a repeated leaching is performed with only clean fresh solvent during 17 min with the a valve adjustment that forces the condensate directly back into the extraction tube. Finally, the level of the solvent is lowered to concentrate the extract. Extractions were performed in triplicate and the mean values were reported.

### 2.5. Soxhlet Extraction Procedure

The sample is transferred to a 33 mm × 100 mm cellulose thimble and placed after in the extraction chamber of a 200 mL capacity Soxhlet apparatus. The cellulose thimble was clogged with cotton in order to avoid transfer of sample particles to the distillation flask. The Soxhlet apparatus, fitted with a condenser, was placed on a 500 mL distillation flask containing 300 mL of solvent and 3 boiling glass regulator. Samples were thus extracted under reflux with dichloromethane during 3 hours (5–6 cycles/h).

After the extraction, the major solvent was eliminated in a vacuum rotary evaporator. The content was then transferred in a smaller tarred flask and concentrated to dryness with a vacuum rotary evaporator. The flask was then weighed and the operation repeated during 30 min until difference between two consecutive weights was smaller than 10% (w/w). Extractions were performed at least three times and the mean values were reported.

Results obtained were expressed as described hereinafter:

$$\%\text{quinoline alkaloids}=\frac{\text{Weight of quinoline alkaloids obtained after}\;\text{extracion}}{\text{Weight of dry sample}}\times 100$$

### 2.6. Analytical Procedure

Reference standards and sample extracts prepared by MIEL or Soxhlet extraction were dissolved in the mobile phase at the concentration of 2 mg/mL. The solutions obtained were subjected to a slight heating when necessary and filtered through a 0.45 μm membrane before injection.

The HPLC system (Shimadzu, Japan) consisted of a LC-10A pump connected to a SPD-10A UV-VIS detector. Analysis of injected extracts was performed with the Start Chromatography Workstation software (version 4.51, Varian, USA). Manual injections were carried out using a Rheodyne injector 77,251 with 20 μL sample loop.

The separation was performed at room temperature on a Microsorb-MV C8 column (5 μm, 250 × 4.6 mm ID, Varian, USA) under isocratic reversed-phase conditions. A flow-rate of 1.4 mL/min was used with a detection wavelength of 316 nm. The elution system was prepared as described below. 700 mL of buffer consisting of 50 mM KH_2_PO_4_ and 30 mM hexylamine were acidified to pH 2.8 with phosphoric acid 10% and then supplemented with 60 mL acetonitrile. 1000 mL of mobile phase was finally obtained by addition of water.

### 2.7. Data Treatment

The investigation and optimization of the performance of the Microwave integrated extraction and leaching (MIEL) was obtained by the response surface methodology (RSM). A Box Wilson procedure, commonly called central composite design (CCD), was used to evaluate the relevance of the three controlled factors (namely extraction time, leaching time and irradiation power). The multivariate study allows the identification of interactions between variables and provides a complete exploitation of the experimental domain to be studied with a reduced number of experiments. The CCD comprise a two-level full factorial design (coded ±1), superimposed by centre points (coded 0) and “star points” (coded ± *α*). The group of “star points” axial experiments located at a distance *α* from the centre, allow rotatability. They also establish new extremes for the low and high settings for all factors, allow estimation of experimental error and provide estimation of the curvature for the model. The precise value of *α* depends on the number of factors involved and on certain properties desired for the design. A CCD can be represented by a cube where each factors corresponds to an axis

The three key variables studied were pointed at five separate coded levels: −*α* (=−1.68), −1, 0, +1, +*α* (=1.68) and their values were selected on the basis of previous experiments. The natural values and coded levels used in this multivariate study are presented in Table 1.

**Figure 2:**
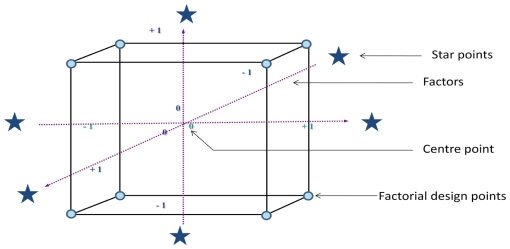
Distribution of the experimental points in a three variable central composite design.

This complete procedure involves 20 experiments including six replications of the center points to take the experimental error of the measurements into account, thus allowing isovariance estimation. The distribution of the experimental points can be displayed on a graphical representation (see Figure 2). The experiments were randomized to prevent effects of extraneous variables. The surfaces responses and interpretations of data obtained were analyzed by the statistical experimental design computer program Statgraphics Plus (2000).

## 3. Results and Discussion

### 3.1. Central Composite Design Results

Three variables that affect extraction of quinoline alkaloids from bark of *C. succirubra* were studied: namely, “extraction time”, “leaching time” and “microwave irradiation power”. These key variables were involved in a central composite design in order to evaluate, optimize and conduct relevant microwave-assisted extraction of quinoline alkaloids from bark of *C. succirubra*. Microwave irradiation power ranged from 60 W to 240 W. The chosen power limits were function of solvent use and function of regulation limitations in the microwave apparatus. The extraction time and leaching time range chosen (from 5 to 19 min) were relatively short yet competitive with conventional extraction. These three controlled variables were studied in a multivariate study with 20 experiments as shown in the Table 2, were triple coded values and yield obtained in the multivariate study for each experiment are described.

Table 1 shows the independent variables, the 5 levels used and the experimental design in terms of coded and uncoded data. Table 2 shows the 20 experimental point runs according to the MIEL experimental planning. The relative yield of quinoline alkaloids obtained by MIEL ranged from 2.42% to 4.04%. The experimental data was analyzed using response surface regression procedure using Statgraphics Plus^®^ to obtain the predicted model for the extraction yield of quinoline alkaloids from bark of *C. Succirubra* and the subsequent optimized extraction conditions.

Experimental data allowed us to fit the yield of extracted as a function of extraction time, leaching time and applied power. The second-order polynomial equation of the response surface obtained is represented as follows:

Yield (%): 10.2505 − 0.4637*T* − 0.1794*L* − 0.0399*P* + 0.0123*T^2^* + 0.0106*L^2^* + 0.0001*P^2^* − 0.0014*TL* + 0.0013*TP* − 0.0007*LP*, where *T* denotes extraction time (min), *L* the leaching time (min) and *P* is the irradiation power (W).

An analysis of variance (ANOVA) was carried out in order to test the model signification and suitability. Thus, various statistical data such as standard error, sum of squares, *F*-ratio or *p*-value are given in ANOVA (Table 3).

**Figure 3:**
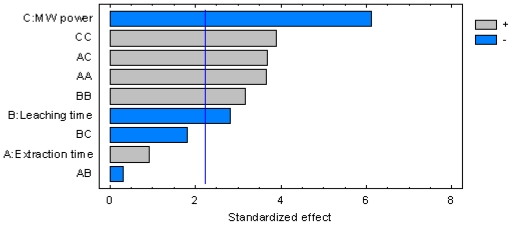
Standardized Pareto chart.

The *F*-ratio in this table is the ratio of the mean-squared error to the pure error obtained from the replicates at the design center. The significance of the *F*-value depends on the number of degrees of freedom (Df) in the model and is shown in the *p-*value column (95% confidence level). Thus, the effects lower than 0.05 in this column are significant. A Pareto chart of standardized effects (Figure 3) was carried out in order to show significant effects of all variables (linear, quadratic and interactions between variables).

The length of the bars is proportional to the absolute magnitude of the estimated effects coefficients while the dashed line represents the minimum magnitude of statistically significant effects (95% of the confidence interval) with respect to the response. It can be seen that microwave power has the most important influence on yields followed by squared term of power and extraction time, interaction of extraction time and power, square root of leaching time, and the leaching time.

### 3.2. Optimal Conditions

Response surface optimization can be calculated depending on the three key variables, namely extraction time, leaching time and power. The maximum yield computed by the software is calculated by the maximum value of the surface response for a set of variables lying between the minimum and maximum value of the CCD plan. The optimized conditions obtained using the model for each parameter were: extraction time, 5 min; leaching time 17 min and irradiation power 60 W, 60 W. Using those optimized conditions, the model predicted a maximum response of 5.5%. In order to verify the predicted response and assure the validity of the results, an experiment using optimized conditions was carried out in triplicate and compared to the statistically predicted value. A mean value of 5.4 ± 0.1% of yield obtained from MIEL experiments validated the reponse surface methodology model, representing a comparable yield if compared to conventional Soxhlet extraction (5.7%). Therefore, the MIEL technology represents a promising alternative to Soxhlet extraction with comparable yields in significantly shorter time with reduced use of solvents.

### 3.3. Kinetics of Extraction: Comparison of MIEL vs. Soxhlet

The yield of quinoline alkaloids obtained from *C. succirubra* was 5.65 ± 0.07% and 5.7 ± 0.09% (w/w) for the MIEL (5 min, 17 min, 60 W) and the conventional Soxhlet (3 h) respectively.

**Figure 4:**
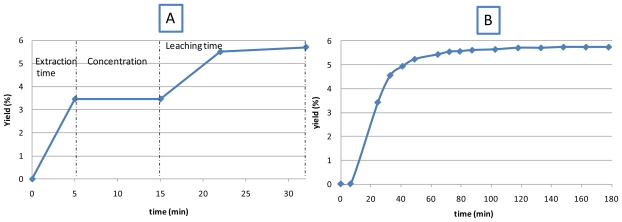
Comparison of kinetics of extraction realized by MIEL (**A**); and by conventional Soxhlet (**B**).

Figure 4 shows the variation of the extraction yield according to the extraction time and the three observed phases in the process of microwave extraction.

The first step (0 to 5 min) is represented by an increasing line which characterizes the extraction time. This phase is followed by the second step, where the level of the solvent is lowered below the sample. In this stage (realized into 10 min) the quinoline alkaloids amount represents nearly 60% of the global yield. The third part corresponds to the leaching time and after 17 min we obtained the maximum of the yield (5.7%).

What was initially observed is that MIEL increased the kinetic of extraction. We can see that during the extraction time we obtained a yield of 3.46% by MIEL in 5 min whereas 25 min are needed to reach this performance using the conventional method.

### 3.4. Analysis of Principal Alkaloids

**Figure 5:**
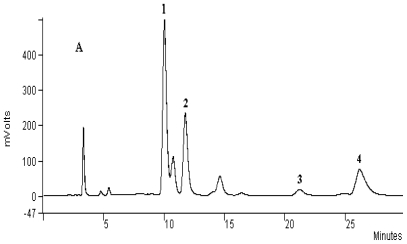

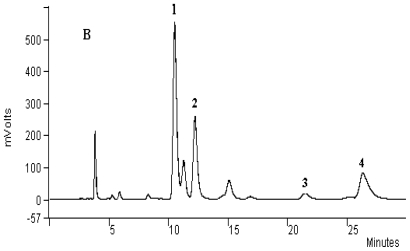
Comparison of HPLC chromatograph profiles of extraction realized by MIEL (**A**) and by conventional Soxhlet (**B**), separation of alkaloids on the Microsorb-MV C8 column. Peaks: 1 = cinchonine, 2 = cinchonidine, 3 = quinidine, 4 = quinine.

The chromatograph corresponding to the *Cinchona* alkaloids is presented in Figure 5.

**Figure 6:**
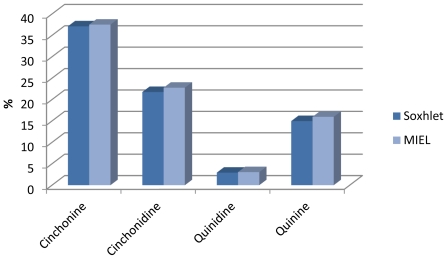
Composition of extract.

The relative percentages of the different compounds are calculated by internal normalization by comparing samples against previously injected standards (Figure 6).

The quinoline alkaloids extracts provided either by MIEL or Soxhlet contain the same dominant components. Cinchonine, cinchonidine and quinine were the three major compounds in the extract.

The results by MIEL are similar to those obtained by Soxhlet. Extract composition was equivalent in terms of quinine (~15%), quinidine (~2.9%), cinchonidine (~22%) and cinchonine (~37%) for both extraction methods but also in terms of yield (5.7%).

In this application, microwave irradiation highly accelerated the extraction process (32 min), but without causing considerable changes in the composition; a phenomenon which was already described by Paré *et al.* [31,32] and Chemat *et al.* [33].

It is important to note that microwave extraction with a long operating time can lead to degradation of valuable products as reported by Cañizares-Macías *et al*. [34]. This phenomena is not due to microwave energy which is very low compared to energy bonds (covalent, hydrogen, Van der Waals or Brownien motion) but it is probably due to an intense heating and an over boiling leading to an enhancement of 10 to 40 °C of the extraction temperature. These phenomena have been reported by Chemat and Esveld [35] and clearly observed while boiling a variety of protic and aprotic solvents, affecting the kinetics of degradation reactions through Arrhenius equation, by increasing the temperature of extraction and reaction.

In this paper we try to optimize our experimental conditions by reducing the extraction and leaching time in order to prevent this degradation phenomenon. The analysis of the extracts do not show a substantial difference between conventional and microwave extracts, but this does not mean that there is no degradation at ppm or ppb level.

### 3.5. Cost, Energy, and Environmental Ecology

MIEL is proposed as an “environmentally friendly” extraction method for quinoline alkaloids. The reduced cost of extraction is clearly advantageous for the proposed MIEL method in terms of energy, solvent used and time. Conventional procedure required an extraction time of 3 hours. The MIEL method required heating for only 32 min. The energy required to perform the two extraction methods are respectively 3 kW·h for conventional Soxhlet (electrical energy for heating and evaporating) and 0.15 kW·h for MIEL (electrical energy for microwave supply). The power consumption has been determined with a Wattmeter at the microwave generator entrance and the electrical heater power supply. Concerning environmental impact, the calculated quantity of carbon dioxide rejected in the atmosphere is higher in the case of conventional Soxhlet extraction (2823 g CO_2_/g of quinoline alkaloid extract) than for MIEL (148 g CO_2_/g of quinoline alkaloid extract). These calculations have been made according to literature: to obtain 1 kW·h from coal or fuel, 800 g of CO_2_ will be rejected in the atmosphere during combustion of fossil fuel [36].

## 4. Conclusions

The usefulness of the MIEL process for quinoline alkaloids extraction from bark of *C. succirubra* has been studied. This original device combining microwave extraction and leaching provides a valuable alternative for quinoline alkaloids extraction. The efficiency of the MIEL method is considerably higher than the conventional Soxhlet procedure, especially in terms of shortening extraction time, reduction of solvent used by recycling during extract concentration, energy consumption and cleanliness of the process. It also reduces toxic releases and saves energy consumption which can lead to climate change and/or greenhouse gas emission benefits.

## Figures and Tables

**Table 1:** Coded levels and natural values applied to the three factors in the experimental design.

Level	Extraction Time (min)	Leaching Time (min)	MW Power (W)
−α (−1.6818)	5	5	60
−1	8	8	100
0	12	12	150
+1	16	16	200
+α (1.6818)	19	19	240

Extraction time = time for solid-liquid contact to ensure the transfer of solute; Leaching time = time for rinsing solid material with fresh solvent.

**Table 2:** Fully coded central composite design and responses obtained.

Run Order	Extraction Time (min)	Leaching Time (min)	MW Power (W)	Response (%)
1	0 (12)	1.6818 (19)	0 (150)	3.03
1	0 (12)	1.6818 (19)	0 (150)	3.03
2	0 (12)	0 (12)	0 (150)	2.72
3	0 (12)	0 (12)	0 (150)	2.71
4	1 (16)	1 (16)	1 (200)	2.85
5	−1 (8)	1 (16)	1 (200)	2.42
6	−1.6818 (5)	0 (12)	0 (150)	2.91
7	0 (12)	0 (12)	0 (150)	2.74
8	0 (12)	0 (12)	1.6818 (240)	2.84
9	−1 (8)	−1 (8)	1 (200)	3.1
10	−1 (8)	1 (16)	−1 (100)	4.04
11	1.6818 (19)	0 (12)	0 (150)	3.73
12	−1 (8)	−1 (8)	−1 (100)	3.97
13	0 (12)	0 (12)	−1.6818 (60)	3.87
14	0 (12)	0 (12)	0 (150)	2.71
15	1 (16)	−1 (8)	−1 (100)	3.43
16	0 (12)	0 (12)	0 (150)	2.8
17	0 (12)	0 (12)	0 (150)	2.75
18	1 (16)	1 (16)	−1 (100)	3.18
19	1 (16)	−1 (8)	1 (200)	3.39
20	0 (12)	−1.6818 (5)	0 (150)	3.46

**Table 3:** Summary of the ANOVA model statistics.

Effect	Sum of Squares	Df	Mean Square	*F*-Ratio	*p*-Value
A:Extraction time	0.0357836	1	0.0357836	0.87	0.3737
B:Leaching time	0.330082	1	0.330082	8.00	0.0179
C:MW power	1.54419	1	1.54419	37.42	0.0001
AA	0.556121	1	0.556121	13.47	0.0043
AB	0.00405	1	0.00405	0.10	0.7605
AC	0.5618	1	0.5618	13.61	0.0042
BB	0.416118	1	0.416118	10.08	0.0099
BC	0.1352	1	0.1352	3.28	0.1004
CC	0.628391	1	0.628391	15.23	0.0030
Total error	0.412711	10	0.0412711		
Total (corr.)	4.36238	19			

*R*^2^ = 0.9054.
